# Collaborative care for panic disorder, generalised anxiety disorder and social phobia in general practice: study protocol for three cluster-randomised, superiority trials

**DOI:** 10.1186/s13063-017-2120-3

**Published:** 2017-08-16

**Authors:** Nadja Kehler Curth, Ursula Ødum Brinck-Claussen, Annette Sofie Davidsen, Marianne Engelbrecht Lau, Merete Lundsteen, John Hagel Mikkelsen, Claudio Csillag, Carsten Hjorthøj, Merete Nordentoft, Lene Falgaard Eplov

**Affiliations:** 1 Mental Health Center Copenhagen, Mental Health Services, Capital Region of Denmark, Kildegårdsvej 28, 2900 Hellerup, Denmark; 2 Research Unit for General Practice and Section of General Practice, University of Copenhagen, Øster Farimagsgade 5, PO Box 2099, 1014 Copenhagen K, Denmark; 3 Stolpegård Psychotherapy Centre, Mental Health Services, Capital Region of Denmark, Stolpegårdsvej 20, 2820 Gentofte, Denmark; 4 Mental Health Center Frederiksberg, Mental Health Services, Capital Region of Denmark, Nordre Fasanvej 57-59, 2000 Frederiksberg, Denmark; 5 Mental Health Center North Zealand, Mental Health Services, Capital Region of Denmark, Dyrehavevej 48, 3400 Hillerød, Denmark; 6 Institute for Clinical Medicine, University of Copenhagen, Mental Health Center Copenhagen, Mental Health Services, Capital Region of Denmark, Kildegårdsvej 28, 2900 Hellerup, Denmark; 7 Independent General Practitioner, Copenhagen, Denmark

**Keywords:** Collaborative care, Shared care, Anxiety disorders, Panic disorder, Generalised anxiety disorder, Social phobia, Cluster-randomised trial, General practice

## Abstract

**Background:**

People with anxiety disorders represent a significant part of a general practitioner’s patient population. However, there are organisational obstacles for optimal treatment, such as a lack of coordination of illness management and limited access to evidence-based treatment such as cognitive behavioral therapy. A limited number of studies suggest that collaborative care has a positive effect on symptoms for people with anxiety disorders. However, most studies are carried out in the USA and none have reported results for social phobia or generalised anxiety disorder separately. Thus, there is a need for studies carried out in different settings for specific anxiety populations.

A Danish model for collaborative care (the Collabri model) has been developed for people diagnosed with depression or anxiety disorders. The model is evaluated through four trials, of which three will be outlined in this protocol and focus on panic disorder, generalised anxiety disorder and social phobia. The aim is to investigate whether treatment according to the Collabri model has a better effect than usual treatment on symptoms when provided to people with anxiety disorders.

**Methods:**

Three cluster-randomised, clinical superiority trials are set up to investigate treatment according to the Collabri model for collaborative care compared to treatment-as-usual for 364 patients diagnosed with panic disorder, generalised anxiety disorder and social phobia, respectively (total *n* = 1092). Patients are recruited from general practices located in the Capital Region of Denmark. For all trials, the primary outcome is anxiety symptoms (Beck Anxiety Inventory (BAI)) 6 months after baseline. Secondary outcomes include BAI after 15 months, depression symptoms (Beck Depression Inventory) after 6 months, level of psychosocial functioning (Global Assessment of Functioning) and general psychological symptoms (Symptom Checklist-90-R) after 6 and 15 months.

**Discussion:**

Results will add to the limited pool of information about collaborative care for patients with anxiety disorders. To our knowledge, these will be the first carried out in a Danish context and the first to report results for generalised anxiety and social phobia separately. If the trials show positive results, they could contribute to the improvement of future treatment of anxiety disorders.

**Trial registration:**

ClinicalTrials.gov, ID: NCT02678624. Retrospectively registered 7 February 2016; last updated 15 August 2016,

**Electronic supplementary material:**

The online version of this article (doi:10.1186/s13063-017-2120-3) contains supplementary material, which is available to authorized users.

## Background

Across surveys, anxiety disorders are found to be the most prevalent group of mental disorders in the general population, with an estimated lifetime prevalence of 16% on average for any anxiety disorder [1]. Among patients with anxiety disorders, there is a high level of comorbidity with other psychiatric disorders such as other anxiety disorders, depression and alcohol abuse [2–4], but also physical illnesses [5, 6]. The human costs related to anxiety disorders are high and place a significant burden on the economy due to high levels of health care use, lost income and sick leave. In Denmark, the total cost related to anxiety disorders is estimated to be six billion DKK per year [7], approximately US$900 million.

The majority of patients with anxiety disorders in Denmark are treated in general practice [8]. For general practitioners (GPs), people with anxiety disorders represent a significant part of their patient population as they use health care services more frequently compared to patients without anxiety disorders [9–11]. It is estimated that 1–7% of the patients in general practice have panic disorder [12–14], 4–12% have generalised anxiety disorder [3, 12, 13, 15] and 4–6% have social phobia [3, 13].

Patients with non-psychotic disorders, such as anxiety disorders, are poorly recognised and often not treated sufficiently [3, 15–22]. The main obstacles for adequate treatment are poor coordination between sectors and a lack of competent treatment availability in general practice [23]. Cognitive behavioural therapy (CBT) is an evidence-based treatment for anxiety disorders and recommended in treatment guidelines [19, 24]. In Denmark, GPs can refer patients either to an independent psychologist through an existing public scheme, which includes up to 24 sessions with partly subsidised fees for patients between 18 and 38 years, or a public insurance-paid independent psychiatrist, where they often have to wait for months due to long waiting lists [23]. GPs can offer a limited number of sessions of psychotherapy, but only if they receive supervision [25]. It is estimated that only around one third of GPs fulfil these requirements [23, 26–28]. Furthermore, there is no training in psychotherapy in GPs’ specialist training and no requirements for the GP to participate in postgraduate training to improve their skills and ensure that they are continuously updated and provide a high quality of care. This treatment gap calls for more accessible evidence-based treatment opportunities in general practice.

Danish guidelines for anxiety disorders and depression suggest that improving the treatment in general practice should be done by introducing shared-care interventions such as collaborative care programs [19, 29]. Collaborative care models for anxiety disorders and depression are complex interventions; however, there are four criteria which should be fulfilled: (1) a multi-professional approach to treatment, (2) scheduled follow-up, (3) enhanced interprofessional communication and (4) treatment according to a structured treatment plan [30].

Studies on collaborative care for anxiety disorders are limited. According to a Cochrane review from 2012, collaborative care between the primary and secondary care system provides a useful addition to clinical pathways for the treatment of anxiety disorders and depression and shows significant improvements in treatment outcomes for up to 2 years compared with treatment-as-usual in general practice [30]. However, the review only included four studies reporting results for panic disorder, generalised anxiety disorder or social phobia and they were all conducted in the USA [31–34]. A recent review updated the evidence base for anxiety disorders [35] and included an additional three studies from Germany and The Netherlands [36–38] and also found collaborative care superior to treatment-as-usual, with a small effect size for all anxiety disorders combined and a moderate effect size in a subgroup of studies on patients with panic disorder. Additional analysis in the review [35] also showed a greater effect size in studies performed in the USA compared to studies performed in Europe in studies that included a care manager and in studies using stepped collaborative care, although this is to be interpreted with caution due to the small number of studies and the heterogeneity between the European studies. Thus, current evidence builds on few studies all conducted in organisational settings not comparable with the Danish or Scandinavian ones. Furthermore, no studies report results for social phobia or generalised anxiety disorder separately. Therefore, there is a need for further research into collaborative care models facilitating a more effective implementation of the intervention as well as for specific anxiety populations in different organisational contexts.

The development of a Danish model for collaborative care (the Collabri model) for panic disorder, generalised anxiety disorder and social phobia was completed in 2014 in collaboration between Danish GPs, psychiatrists and researchers. In this paper the protocol (version 2) for three cluster-randomised controlled, superiority trials investigating collaborative care, according to the Collabri model, vs. treatment-as-usual for panic disorder, generalised anxiety disorder and social phobia will be outlined. As we developed specific collaborative care treatment models for each of the three anxiety disorders, and wanted to examine the effect of each of these three collaborative treatment models, we designed three cluster-randomised controlled, superiority trials; but as the trials for each of three anxiety disorders are similar in terms of aim, design and methods, they are jointly presented in this paper. The hypothesis for each of the trials is that treatments according to the Collabri model are more effective than treatment-as-usual.

## Methods

### Aim and design

**Fig. 1:**
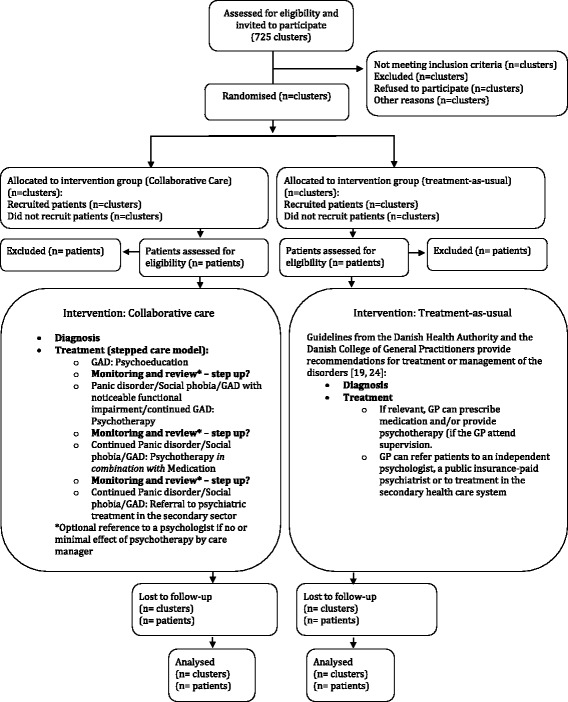
Flow chart for participants. Each trial has a flow chart similar to this

The aim of the three trials is to test if treatment according to the Collabri model is more effective than treatment-as-usual. The null hypothesis for each trial to be rejected is that collaborative care according to the Collabri model (intervention group) and treatment-as-usual (control group) have the same effect on anxiety symptoms for patients with panic disorder, generalised anxiety disorder and social phobia in general practice. The trials are designed as three independent, two-armed, cluster-randomised, clinical superiority trials (see the flow chart in Fig. 1). All aspects of the study design (including assessment time points, outcome measures, etc.) are identical in the three trials. A total *n* = 1092 is required based on sample size calculations; 364 patients in each of the three trials (diagnoses) on panic disorder, generalised anxiety disorder and social phobia, respectively.

### Eligibility criteria for participants

#### Cluster level

GPs with a registered provider number, practicing in the Capital Region of Denmark, are eligible for the trials. For practical reasons, GPs on the island of Bornholm are not eligible. The local branch of the Organisation of General Practitioners in Denmark and the Capital Region of Health Care have negotiated and signed an agreement that allows the GPs to participate in the study and sets out the terms and conditions for it, including financial reimbursement.

#### Individual level

Patients are eligible for the trials if they consult a GP who is participating in the trials and additionally comply with the inclusion and exclusion criteria. These criteria are assessed by the GP at recruitment and/or a research assistant at a baseline eligibility interview.

##### Inclusion criteria

Participating patients should be at least 18 years old, diagnosed in general practice according to the *International Classification of Diseases 10th edition* (*ICD-10*) criteria for panic disorder (F41.0), generalised anxiety disorder (F41.1) or social phobia (F40.1) [39]. They must speak Danish and give their written consent to participation.

##### Exclusion criteria

Patients cannot participate in the trials if they are pregnant, have a pending disability pension application, are at a high risk of suicide, have a current psychotic condition, obsessive compulsive disorder, or bipolar affective disorder, misuse alcohol or other substances to an extent that will hinder the patient’s participation in treatment, if they receive current psychological or psychiatric treatment for depression or anxiety or have been receiving such treatment within the last 6 months. Also, referral to the secondary care system before or at the first GP consultation after baseline eligibility interview, because of a need for more specialised care, is a reason for exclusion. Furthermore, patients cannot participate if it is clinically assessed by the GP that they have dementia or an unstable medical condition that hinders the patient from adhering to treatment. Patients in the intervention group are excluded if they want treatment according to the existing public psychologist scheme and do not want referral to the psychologist to be preceded by treatment according to the Collabri model.

### Exclusion criteriaRecruitment and randomisation

#### Cluster level

The GP recruitment period lasted from May 2014 until July 2015. GP practices were invited to participate via postal letters. This included easy-to-read information about the project and an invitation to attend information meetings. Eligible GPs were randomised to either provide treatment according to the Collabri model or treatment-as-usual. A cluster corresponds to a GP provider number which may include one or several GPs. Cluster-randomisation was chosen, as a significant risk of bias was identified in the form of contamination if randomisation was performed on patient level. Randomisation was performed externally by The Research Centre for Prevention and Health (RCPH) in the Capital Region of Denmark, which used a centralised computer-generated allocation sequence. The randomisation was conducted in two phases. The first included 49 GPs and was stratified by three geographically defined uptake areas. The second randomisation included four GPs, and here a geographical stratification was not used.

#### Individual level

As the trial is randomised on general practice level, the participating patients are allocated to either the intervention or control group according to their GP. The GPs identify and recruit the patients with anxiety disorders. In Danish medical guidelines GPs are recommended to use the Anxiety Symptom Scale (ASS) in conjunction with *ICD-10* criteria for diagnostic investigation [24]. The GP provides written and verbal information about the project, and hands out the Consent Form. Following recruitment, a research assistant contacts the patient to schedule a telephone baseline eligibility interview. The aim is to assess inclusion and exclusion criteria and verify the GP’s diagnosis by conducting a diagnostic interview. If the diagnosis according to the diagnostic interview differs from the diagnosis given by the GP, the research assistant consults a psychiatrist in the Collabri team who contacts the GP in order to discuss and agree on the diagnosis. Patient recruitment was initiated in November 2014 and ended on 31 December 2016.

### Blinding

The research team (who assess baseline eligibility criteria, collect interviewer-based baseline and follow-up assessments, perform statistical analyses and draw conclusions) are blinded to allocation throughout the entire trial period (from baseline eligibility interview to drawing conclusions). It is not possible to blind GPs, the Collabri team who deliver the intervention, the administrative project team and the participating patients towards intervention allocation. It will be highlighted to them that they cannot reveal their allocation group to the research team. Should the allocation be revealed to the research assistant before assessments (baseline eligibility interview or interviewer-based assessments), another research assistant is allocated. Furthermore, during statistical analyses, the two groups will be coded and anonymised (e.g., X and Y) until final analyses and conclusions have been made.

The referral diagnosis is not revealed to the researchers conducting the baseline eligibility interview until the end of the interview. This makes the assessment of baseline eligibility criteria more objective.

### Interventions

#### The experimental intervention – the Collabri model for collaborative care

The Collabri model is developed based on the previously mentioned four criteria for collaborative care [30] and has been adapted to Danish conditions by including collaboration with relevant professionals in the local authorities and integrating the existing public psychologist scheme. In addition, the model integrates elements which are found to be essential for collaborative care interventions in an academic literature review [40] or recommended in current guidelines [19, 24, 41] recommending the use of screening instruments for detection and follow-up, and a stepped-care approach to treatment. In addition, the model incorporates principles of involvement of relatives, patient involvement and influence on treatment and support in self-management. Below, how the Collabri model meets the four criteria for collaborative care is outlined.

The model includes a multi-professional approach to treatment involving a GP, a care manager and a psychiatric specialist. Interprofessional communication consist of weekly meetings between the GP and care manager in order to discuss clinical cases and of the psychiatric specialist supervising the care managers in groups twice a month and the GPs in groups once a month. When needed, the GP, care manager and the psychiatric specialist have joint consultations and the GPs and care managers receive individual supervision from the psychiatric specialist if required. The weekly meetings between care manager and GP take place in person, but other communication can take place via video conferences if it is not possible to meet in person.

It has not been possible to establish a joint recording system between primary and secondary care systems; however, written communication between the GP and care manager or psychiatric specialist occurs through already existing electronic communication systems.

All patients commence their treatment by developing an individually structured treatment plan which is based on disorder-specific manuals (unpublished, available through the corresponding author) following the stepped-care principle and complying with national guidelines [19, 24], see Fig. 1. The core treatment elements of the model are: disorder-specific written material and a self-management book (bibliotherapy), CBT, group-based or individual manualised psychoeducation based on the Chronic Disease Management Program (CDSMP)) or as a part of CBT, individual CBT (10–12 sessions, depending on disease) and/or medication. The treatment period is 3–4 months. The number of sessions in the Collabri group will be obtained and reported.

Depending on the disorder and severity, the patients will be offered treatment elements according to a stepped-care plan, offering the least invasive and least resource-demanding treatment first (stepwise scaling up of treatment efforts) (see Fig. 1).

The care manager ensures active and scheduled follow-up including regular monitoring and review of progression. Monitoring takes place as a minimum every 2 weeks or more often depending of the severity of the disorder or when medication is initiated. Regular reviews of the treatment plan will take place at least once a month, and must precede stepping up as well as ending treatment.

##### The multi-professional Collabri team

In the Collabri treatment, the GP works in combination with the Collabri team (the care manager and the psychiatrist/psychologist) around the treatment of the patient). The GP has the overall treatment responsibility, including the diagnostic process, initiates the treatment and collaborates with the care manager to provide seamless treatment and care.

The Collabri team is a health care team consisting of care managers and psychiatric specialists. The psychiatric specialists can either be psychiatrists or psychiatrists in combination with a psychologist with specialist training in psychiatry. The care managers have a medium-long health professional education; for example, as a nurse with mental health care experience as well as being certified in providing CBT (the minimum requirement is a 1-year recognised course). Care managers, GPs and the psychiatrists ensure adherence to the Collabri model.

##### Training prior to intervention

All care managers attend a 1-week course introducing them to the Collabri model and the CBT methods. In addition they attend a 4-day training course leading to certification in a manualised group-based psychoeducation program (based on the Chronic Disease Management Program (CDSMP)), and an additional 2-day course of introduction in an individualised psychoeducation manual based on the CDSMP principles. The psychiatric specialists also attend the training except the group psychoeducation training. All GPs in the intervention group participate in a 1-day training course in the principles of collaborative care and the Collabri model.

##### Fidelity

Fidelity assessments ensure that the Collabri intervention is carried out according to the described Collabri model. Assessors will monitor fidelity to the model 6 months after study initiation and at least once more during the inclusion period. Fidelity will be monitored using a Collabri fidelity scale (unpublished, available through the corresponding author). In order to improve implementation, an action plan will be developed where needed based on the outcome of the fidelity assessments.

#### The control intervention – treatment-as-usual

Participants whose GP is randomised to the control group will receive treatment-as-usual as offered by their GP. Guidelines from the Danish Health Authority and The Danish College of General Practitioners are available for guidance [19, 24]. According to the guidelines a physical health evaluation is performed prior to diagnosis and the ASS instrument in conjunction with *ICD-10* criteria is recommended for detection, diagnostic investigation and monitoring of the disorder [42]. If relevant, the GP can prescribe medication and/or provide psychotherapy if they attend supervision. The GP can also refer patients to an independent psychologist (partly publicly subsidised) or to a public psychiatrist or to treatment in the secondary health care system (fully publicly subsidised). Accessible treatment can vary between general practices as these guidelines only provide recommendations for treatment or management of the disorders, as opposed to required minimum standards.

### Assessments and outcome measures

#### Baseline eligibility interview

At baseline, patients attend a telephone eligibility interview where the inclusion and exclusion criteria are assessed by a research assistant. To confirm the diagnosis the *Diagnostic and Statistical Manual of Mental Disorders, 4th edition* (*DSM-IV*) MINI International Neuropsychiatric Interview [43] is used including *ICD-10*-specific questions, for the inclusion diagnoses. The GP assesses comorbidity at recruitment. Information on personality disorder traits are obtained through the Standardised Assessment of Personality: Abbreviated Scale (SAPAS) [44].

#### Outcome measures

**Fig. 2:**
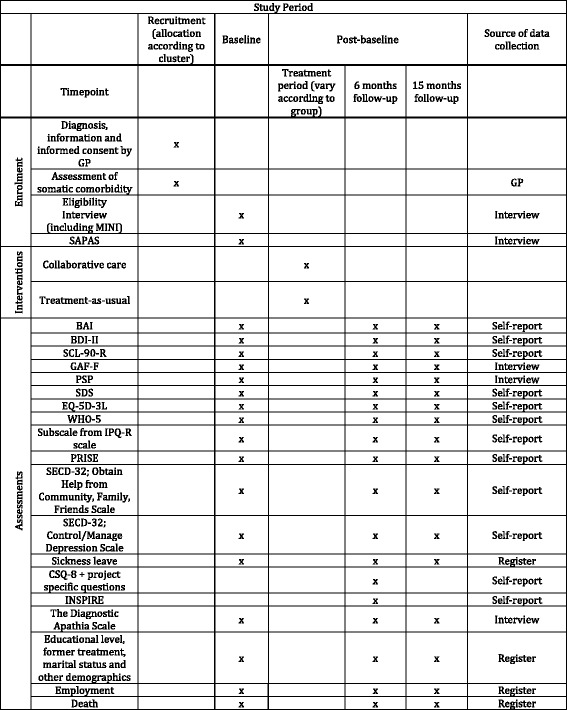
Time schedule, assessment instruments and source of data collection

Participants are interviewed and asked to fill out a questionnaire at baseline, 6 and 15 months’ follow-up. In the following each assessment instrument will be described. See the Standard Protocol Items: Recommendations for Interventional Trials (SPIRIT) figure (Fig. 2) for an overview of time schedule, assessment instruments and source of data collection.

##### Primary outcome measure

The primary outcome is anxiety symptoms measured with the Beck Anxiety Inventory (BAI) 6 months after baseline. The BAI is a 21-item general questionnaire for anxiety, measuring symptoms during the last week rated on a four-point Likert-scale from 0 (never) to 3 (almost all the time), where the maximum score is 63 [45]. The BAI has shown excellent psychometric properties, with internal consistency: *α* = 0.92 and 1-week test-retest reliability: *r* = 0.75 in a community sample [46].

##### Secondary outcome measures

The secondary outcome measures are the BAI at 15 months, depression symptoms (Beck Depression Inventory (BDI-II)) [47] at 6 months, level of psychosocial functioning (Global Assessment of Functioning (GAF-F)) [48] and general psychological symptoms (Symptom Checklist (SCL-90-R)) [49] at 6 and 15 months after baseline.

The BDI-II is a 21-item general questionnaire for measuring depression symptoms during the last 14 days rated on a four-point Likert-scale from 0 to 3, where the maximum score is 63. A meta-analysis of the BDI II’s internal consistency estimates yielded a mean coefficient alpha of 0.86 for psychiatric patients and 0.81 for non-psychiatric subjects [50]. The test-retest reliability for the BDI II for psychiatric patients has been reported to range from 0.48 to 0.86, and for non-psychiatric subjects from 0.60 to 0.83. The SCL-90-R is the revised version of SCL-90, a multi-dimensional questionnaire consisting of 90 questions about general psychological symptoms. The questionnaire consists of nine subscales from which a joint measure (Global Severity Index (GSI)) can be calculated. The internal consistencies of the SCL-90-R are satisfactory with range from *α* = 0.74 (‘aggression’) to *α* = 0.97 (GSI) [51].

The split version of the GAF on psychosocial functioning ranges from 0 to 100 points, with a higher number indicating a higher level of functioning. Studies suggest a good validity of the GAF-F scale as well as usefulness of measuring the level of psychosocial functioning among incident patients with schizophrenia. A high inter-rater reliability can be achieved with little training of the raters [52].

##### Explorative outcome measures

The explorative outcome measures are the BDI-II at 15 months, personal and social performance (PSP) [52], health-related quality of life (EQ-5D-3 L) [53], functional impairment (Sheehan Disability Scale (SDS)) [54], quality of life (WHO-five Well-being Index (WHO-5)) [55] and self-efficacy (Personal Control subscale from the revised version of the Illness Perception Questionnaire (IPQ-R) [56] and two subscales from the Chronic Disease Self-Efficacy Scales (SECD-32); and the Obtain Help from Community, Family, Friends Scale and Control/Manage Depression Scale) [57] at 6 and 15 months.

Here, a measure of apathy will also be collected through the Diagnostic Apathia Scale, which consists of six items [58] and side effects from treatment will be measured with the Patient-Rated Inventory of Side Effects (PRISE) which identifies and evaluates the tolerability of symptoms/side effects in nine domains [59].

The PSP measures personal and social functional level in the domains of: socially useful activities (for example, work and education), personal and social relationships, self-care and disturbing and aggressive behaviours on a scale from 1 to 100 with a higher number indicating higher level of function.

The EQ-5D-3 L is a measure of health status in five domains: mobility, self-care, usual activities, pain/discomfort and anxiety/depression and also includes a Visual Analogue Scale from 0 (worst imaginable health status) to 100 (best imaginable health status).

The SDS is a composite of three items designed to measure the extent to which three major domains in the patient’s life are impaired by symptoms, and can be summed into a joint measure of global functional impairment that ranges from 0 (unimpaired) to 30 (highly impaired). The WHO-5 consists of five items that measure the subjective experience of quality of life or psychological wellbeing. Each item is rated on a six-point Likert scale from 0 (not present) to 5 (constantly present).

The IPQ-R has 12 subscales of which the Personal Control subscale consists of six items about one’s own beliefs about the ability to affect the disorder. Each item is rated on a five-point Likert-scale from 1 (disagree very much) to 5 (agree very much).

The two subscales of SECD-32 consist of four and six items about how confident one is in doing certain activities. Each item is rated on a 10-point Likert scale from one (not at all confident) to 10 (very confident).

Information about life-threatening conditions, the use of inpatient and outpatient physical and mental health services and number of days of admission and former treatment is collected through the National Patient Registry which holds information about all patient contacts in the secondary health care system [60].

Medication use is retrieved from the Danish National Prescription Registry which holds information about all sales of medication [61].

Information about deaths is collected through The Danish Register of Causes of Death, which is based on information of all deaths in Denmark since 1943 [62].

Sickness leave, employment and use of other social services will be obtained from the Danish Register for Evaluation of Marginalisation (DREAM), which contains information about contact with the labour market for the entire Danish population [63]. These data are collected at baseline, 6 and 15 months.

At 6 months the patient’s feeling of being supported in their recovery by their primary health care provider (care manager and GP in the intervention group and GP in the control group) is assessed through the INSPIRE questionnaire, which has two sections – one about support (20 items) and one about relationship (seven items) [64].

Participants also rate their general satisfaction with treatment through the CSQ-8 questionnaire [65] together with project-specific questions. The CSQ-8 consists of eight items which are rated on a scale from 0 to 4.

Baseline information, such as former treatment, education and marital status, will be collected through Statistics Denmark, which is the central authority on Danish statistics [66], and the National Patient Registry.

### Data collection

Interview-based assessments such as the GAF-F and the PSP will be conducted through telephone interviews by research assistants who are thoroughly trained in using the instruments. Other data are collected either through self-reported questionnaires or registers (see Fig. 2 for details of sources of data collection). Self-reported questionnaires are completed online or in a paper version. If participants have not completed the questionnaire after 8 days, they will be reminded and, if necessary, they will be contacted in order to collect the missing data. The BAI and BDI-II data can be collected via telephone if it is not possible for the participants to fill them out by themselves. Information about intervention-specific services and treatment will be registered by the care managers and psychiatrists throughout the intervention period.

### Safety measures

The following safety measures are collected:

- Self-reported anxiety and depression symptoms measured with the BAI [45] and BDI-II [47]
- Suicidal ideation obtained from the questions concerning suicidality in the MINI baseline eligibility interview [43]
- Death (natural, accident, suicide, homicide/violence or unknown) obtained from the Danish Register of Causes of Death [62]
- Life-threatening conditions for reasons other than suicide attempts obtained from the National Patient Registry [60]
- Number of physical and mental health outpatient services, admissions and inpatient days obtained from the National Patient Registry [60]
- Number of sickness leave days obtained from the DREAM database [63]

### Training and inter-rater reliability

Trained research assistants will perform the baseline eligibility interviews as well as the objective assessments. Based on sound records, inter-rater reliability is assessed throughout the training and assessment period and discrepancies are discussed with the other raters and at least one of the intervention developers. In the first 3 months, bi-monthly or monthly meetings are held to discuss potential difficulties in rating the objective measures of the PSP scale and the GAF-F scale. Subsequently, reliability ratings related to these measures are performed approximately every 3 months throughout the assessment period.

### Power and sample size calculation

Differences in clinically relevant treatment response for the primary outcome measure BAI is set at 4 points based on international academic literature for the BDI [67, 68], as academic literature on the BAI could not be found. International academic literature suggests that a standard deviation (SD) of 12 for the BAI can be used in the sample size calculation [36, 68, 69]. The intraclass correlation coefficient (ICC) is set at 0.04 based on a review on ICC for anxiety and depression and other mental disorders in primary care [70]. Sample size calculations based on these numbers show that 364 persons for each trial on panic disorder, generalised anxiety disorder and social phobia should be included in order to reject the null hypothesis that the intervention and control groups have improved their symptom level equally when the power is set at 0.8 and the significance level at 0.05.

Power calculations for the secondary outcomes have been estimated to be more than 0.8 based on calculations with 182 participants in each group (see Table 1). As we have not been able to find estimates for the BDI-II and the GAF-F for patients with anxiety disorders in general practice, estimates for patients with depression in general practice have been used as reference for power calculations for the BDI-II [36], whereas patients with social phobia recruited from psychiatric clinics have been used as reference for power calculations for the GAF-F [71]. Estimates for the SCL-90-R are based on a mixed anxiety and depression population in an outpatient setting [72].

**Table 1:** Power calculations for the secondary outcomes

Secondary outcomes	Mean difference (MD)	Standard deviation (SD) of the pooled mean	Type 1 error	Calculated power
BDI-II	4 [77]	11 [36, 77]	5%	93%
GAF-F	5^a^	11 [71]	5%	99%
SCL-90-R	23 [72]	50 [72]	5%	99%

^a^For the GAF-F the expected mean difference has been conservatively estimated to be 5 points as this is considered clinically relevant

*BDI* Beck Depression Inventory, *GAF* Global Assessment of Functioning, *SCL* Symptom Checklist

### Statistical analyses

Data analyses will be carried out according to the statistical principle ‘intention-to-treat’ [73]; thus, once a person meeting the eligibility criteria is included in the study the person stays in the study population, including follow-up, regardless of whether the person later meets the exclusion criteria. All continuous outcome measures will be analysed using generalised linear models. Multi-level, linear mixed models with repeated measures will be used to handle the cluster-randomisation and unequal loss to follow-up. The models will be estimated using an unstructured covariance matrix if possible. If not possible, other covariance matrices, such as independent, interchangeable, autoregressive, and Toeplitz, will be estimated, and the best fitting structure selected based on Bayes’ information criterion. The analysis levels are: GP, patient and time. This model is based on the assumption that data are missing at random or missing completely at random. Explorative subgroup analyses will be made for patients with somatic comorbidity and personality disorders.

### Feasibility

Twelve months’ prevalence rates, based on a meta-analysis including studies conducted in European countries [74], indicate that a GP with 1600 registered patients will on average see 37 patients with panic disorder, 24 patients with generalised anxiety disorder and 32 patients with social phobia per year. By including a minimum of 48 GPs in each trial we find it realistic to include 364 patients with panic disorder, 364 patients with generalised anxiety disorder and 364 patients with social phobia, adding up to a total of 1092 patients. Thus, each GP should recruit approximately seven to eight patients with each anxiety disorder over a 12-month period. With a conservative caseload of 100 patients per care manager per year (25 at a time) and eight care managers, it should be possible in terms of capacity to include up to 1600 patients in the study during 12 months of which 800 would be in the intervention group.

Because there is a risk of GP dropout it might be necessary to reduce the number of GPs to 44. This affects the sample size calculations for the trials and thereby the number of patients needed to include. Thus, 374 participants are required in each of the three trials and each GP should recruit eight to nine patients with each anxiety disorder.

### Project organisation

The project is led by a steering group which ensures the progress of the research project. A lead project manager will ensure that the general management of the project together with a project manager in charge of implementation of the intervention and a project manager in charge of the research project. Administrative staff members support the project managers. Two PhD students and research assistants perform data collection and analyses.

## Discussion

The design of the trials presents several strengths. The randomisation is carried out externally and is computer based which ensures an adequate allocation sequence and concealment, thus reducing the risk of (cluster) selection bias. In order to further limit the risk of selection bias, data will be analysed according to the intention-to-treat principle and attempts to increase follow-up rates will be made. Also, the trials are randomised at cluster level in an attempt to eliminate the potential risk of contamination otherwise introduced by individual randomisation. This risk could be present where GPs trained in collaborative care principles, and receiving supervision from a project psychiatrist, would not be able to differentiate between intervention and control patients when using their acquired skills and advice from the psychiatrist, which is likely to affect the results, and thereby making it more difficult to detect differences between the intervention and the control group. To ensure that the intervention is provided as intended, we monitor fidelity to the Collabri model at least twice during the intervention period.

The primary outcome is self-reported and patients are not blinded towards allocation, which might result in information bias and possibly overestimation of effects [75]. However, the BAI has been found usable for assessing the severity of anxiety symptoms in patients with anxiety disorders in a general practice setting [76], and other outcome measures will be assessor blinded for allocation; for example, the secondary outcome measure of psychosocial functioning (GAF-F). A limitation, as a result of the cluster-randomisation, may be that patients within a cluster have some similarity and may differ from patients from other clusters. However, sample size calculations including an ICC have been carried out, aiming to reduce this problem. Furthermore, we cannot be sure that all eligible patients are asked to participate by their GP, as patients are not systematically screened for eligibility.

The results of these trials will add to the limited pool of knowledge about collaborative care for patients with anxiety disorders. To our knowledge, they will be the first carried out in a Danish context and the first reporting results for generalised anxiety and social phobia separately.

If the trials show positive results, they could contribute to the improvement of future treatment for patients with panic disorder, generalised anxiety disorder or social phobia in general practice.

### Trial status

Recruitment of participants within the clusters is ongoing and continued until 31 December 2016.

## Additional file

Additional file 1: A word file with A Standard Protocol Items: Recommendations for Interventional Trials (SPIRIT) Checklist is included as ‘Additional file 1’. (DOC 123 kb)

## Abbreviations

BAI: Beck Anxiety Inventory
BDI-II: Beck Depression Inventory II
CBT: Cognitive behavioural therapy
CDSMP: Chronic Disease Management Program
CSQ-8: Client Satisfaction Questionnaire
DREAM: Danish Register for Evaluation of Marginalisation
GAF-F: Global Assessment of Functioning Split Scale for Functional Level
IPQ-R: Illness Perception Questionnaire Revised
MINI: Mini International Neuropsychiatric Interview
PRISE: Patient-Rated Inventory of Side Effects
PSP: Personal and Social Performance
SAPAS: Standardised Assessment of Personality: Abbreviated Scale
SCL-90-R: Symptom Checklist-90-Revised
SDS: Sheehan Disability Scale
WHO-5: WHO-five Well-being Index
